# Transcriptomic Analysis Reveals the Dependency of *Pseudomonas aeruginosa* Genes for Double-Stranded RNA Bacteriophage phiYY Infection Cycle

**DOI:** 10.1016/j.isci.2020.101437

**Published:** 2020-08-06

**Authors:** Qiu Zhong, Lan Yang, Linlin Li, Wei Shen, Yang Li, Huan Xu, Zhuojun Zhong, Ming Chen, Shuai Le

**Affiliations:** 1 Department of Clinical Laboratory Medicine, Southwest Hospital, Army Medical University, Chongqing 400038, China; 2 Department of Clinical Laboratory Medicine, Daping Hospital, Army Medical University, Chongqing 400038, China; 3 Shanghai Institute of Phage, Shanghai Public Health Clinical Center, Fudan University, Shanghai 201508, China; 4 Department of Microbiology, College of Basic Medical Sciences, Army Medical University, Chongqing 400038, China; 5 Medical Center of Trauma and War Injury, Daping Hospital, Army Medical University, Chongqing 400038, China; 6 State Key Laboratory of Trauma, Burns and Combined Injuries, Army Medical University, Chongqing 400038, China

**Keywords:** Biological Sciences, Genetics, Molecular Biology

## Abstract

Bacteriophage phiYY is currently the only double-stranded RNA (dsRNA) phage that infects *Pseudomonas aeruginosa* and is a potential candidate for phage therapy. Here we applied RNA-seq to investigate the lytic cycle of phiYY infecting *P. aeruginosa* strain PAO1r. About 12.45% (651/5,229) of the host genes were determined to be differentially expressed genes. Moreover, oxidative stress response genes *katB* and *ahpB* are upregulated 64- to 128-fold after phage infection, and the single deletion of each gene blocked phiYY infection, indicating that phiYY is extremely sensitive to oxidative stress. On the contrary, another upregulated gene *PA0800* might constrain phage infection, because the deletion of *PA0800* resulted in a 3.5-fold increase of the efficiency of plating. Our study highlights a complicated dsRNA phage-phage global interaction and raises new questions toward the host defense mechanisms against dsRNA phage and dsRNA phage-encoded hijacking mechanisms.

## Highlights

• The infection of dsRNA bacteriophage has a modest impact on host gene expression
• Several differentially expressed host genes are essential for dsRNA phage life cycle
• Anti-oxidative genes are highly expressed to support dsRNA phage infection

## Graphical Abstract

**Figure:**
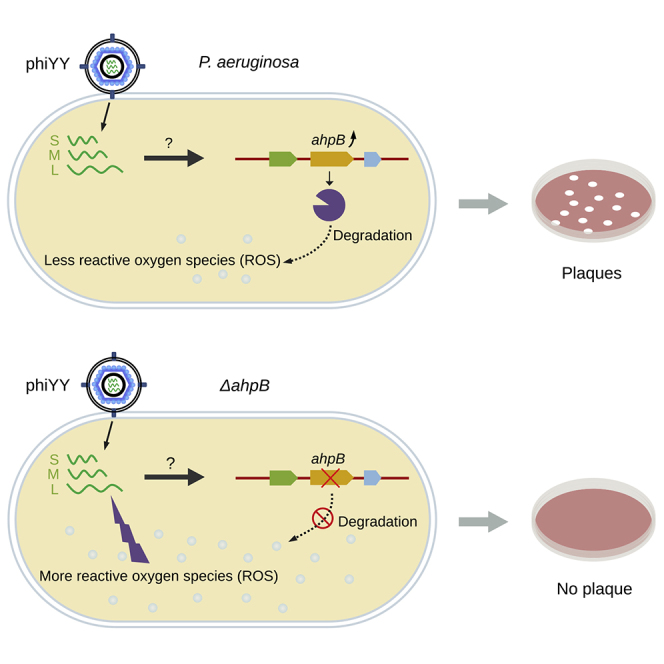

## Introduction

Bacteriophage phiYY is a recently identified member of Cystoviridae (Yang et al., 2016), which have genomes consisting of three double-stranded RNA molecules, L, M, and S. Phage Φ6 was the first identified member of this family isolated in 1973 (Vidaver et al., 1973). Cystoviridae contains three dsRNA segments that are located inside a core particle composed of major structural protein, an RNA-dependent RNA polymerase, a hexameric NTPase, and an auxiliary protein (Nemecek et al., 2010; Mantynen et al., 2018, 2019). The core particle is enclosed within a lipid-containing membrane. However, until now, there are only seven sequenced dsRNA phages yet. And six of them primarily infect *Pseudomonas syringae*, whereas phiYY is the only member that primarily infects *P. aeruginosa* with rough LPS (Yang et al., 2020), which could be included into a phage cocktail to inhibit phage-resistant mutants during phage therapy and might be used to kill the rough LPS strains selected after chronic infection with antibiotic treatment (Hocquet et al., 2016).

*P. aeruginosa* is a common opportunistic pathogen that causes infections of bloodstream, urinary tract, burn wound, and airways of patients with cystic fibrosis (Waters and Grimwood, 2018). Moreover, with the emergence of multidrug-resistant clinical isolates of *P. aeruginosa,* phage therapy has received renewed attention for treating multidrug-resistant bacterial infections (Smith et al., 2017; Waters et al., 2017; Forti et al., 2018; Jault et al., 2018; Bao et al., 2020). Thus, a solid understanding of the phage-host interaction at the molecular level is valuable for the regulation and legislation of phage therapy in the near future (Rohde et al., 2018).

Transcriptomic approach is a powerful tool to study phage-host interactions and has been widely applied in studying phage-host interactions (Lavigne et al., 2013; Chevallereau et al., 2016; De Smet et al., 2016; Zhao et al., 2017; Li et al., 2020; Lood et al., 2020). It leads to a better understanding of the temporal transcriptional schemes, the impact of phage on host genes expression, and the regulatory mechanisms of phages. For example, the transcriptomic data of *P. aeruginosa* phage pap3 and its host revealed that phage early expressed protein Gp70.1 could affect host gene expression, and further experiment demonstrated that Gp70.1 binds to a global regulator Rpos to control host gene expression (Zhao et al., 2016). Phages could also use phage encoded auxiliary metabolic genes to generate additional metabolites to support its replication (Zhao et al., 2017). *P. aeruginosa* phage PAK_P3 significantly depletes bacterial transcripts to facilitate phage replication (Chevallereau et al., 2016). Moreover, giant phage phiKZ could complete its infection cycle without the support from bacterial transcriptional machinery (Ceyssens et al., 2014), whereas another jumbo phage PA5oct relies on the host RNA polymerase to replicate (Lood et al., 2020).

In this study, we report the RNA sequencing (RNA-seq) analysis of dsRNA phage-*P. aeruginosa* interactions, reveal the gene expression patterns of both phage and host during infection cycle, and experimentally demonstrate that several deferentially expressed host genes are essential for phage life cycle. This study would contribute to the understanding of infection dynamics of dsRNA phage.

## Results and Discussion

### RNA-Seq Experiment Design of phiYY Infection

Phage phiYY is effective in infecting the rough strains and has been included in a phage cocktail to limit the emergence of phage-resistance mutant (Yang et al., 2020). PAO1r_8 is a phage-resistant mutant selected after dsDNA phage PaoP5 infection (Shen et al., 2018). PAO1r_8 deleted 341 genes (PA1880-PA2220) and encoded 5,229 ORFs. It lost O-antigen because of the deletion of *galU* gene. It was renamed PAO1r in short in this study and selected as the host strain for RNA-seq analysis (Table S1).

**Figure 1:**
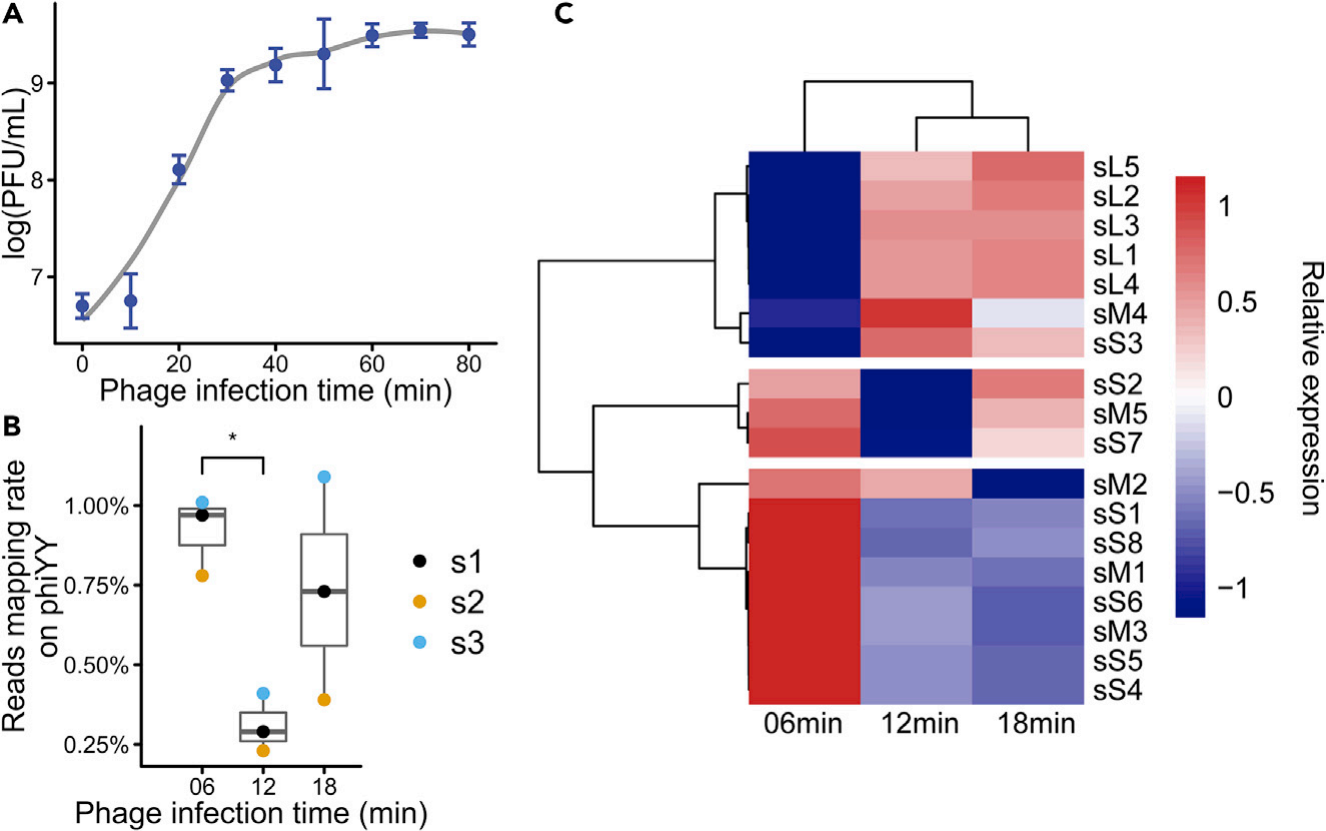
Growth Dynamics of Phage phiYY (A) One-step growth curve of phiYY on PAO1r for 80 min. The latency period is approximately 11 min, and the infection cycle is approximately 18 min. Data are displayed as the means ± standard deviation (error bars) from three independent experiments. (B) Percentage of RNA-seq reads mapped to the phage phiYY genome. Phage reads account for 0.2%–1% of the total reads. S1, S2, S3 represent three biological repeats. (C) Transcriptomic profile of phiYY genes in the infected host. Genes from segment S and M are highly expressed early at 6 min, whereas genes from segment L are highly expressed later at 12 and 18 min.

Next, a one-step growth curve of phiYY infecting PAO1r was measured (Figure 1A). The latency period is approximately 11 min, and the infection cycle duration is approximately 18 min, after which majority of the host are lysed. Thus, we focused on 6 (early), 12 (middle), and 18 min (late) time points after phage was added as representative snapshots of the phage infection cycle.

### dsRNA Phage Transcriptomic Profile

The proportion of reads mapped as phage genes was fluctuating around 0.2%–1% (Figure 1B). This indicates that proportions of dsRNA phage transcripts are quite minor. On the contrary, the mRNA of dsDNA phage could reach a very high percentage. For example, the proportion of phage reads was over 80% and 60% for *Acinetobacter baumannii* phage phiAbp1 (Yang et al., 2019) and *P. aeruginosa* Podovirus LUZ19 (Lavigne et al., 2013), respectively. There are several explanations for the lower phage transcripts. First, dsRNA phages have a very small genome, for example, the sizes of the segments of phiYY were 3,004 (S), 3,862 (M), and 6,648 (L) bp (Yang et al., 2016). On the contrary, the genomes of dsDNA phages usually range from dozens to hundreds of kilo bases (Ceyssens and Lavigne, 2010; Shen et al., 2016). Moreover, dsRNA phages encoded proteins to block bacterial transcriptional machines are not reported yet, whereas these proteins are common in dsDNA phages (Roucourt and Lavigne, 2009). For example, bacteriophage Xp10 encoded protein P7 could bind to the host's polymerase to block host RNA transcription (You et al., 2019).

The phage gene expression pattern can be clustered into early and late expressed genes (Figure 1C). Most genes from segment S and M are highly expressed at 6 min and decrease thereafter, whereas genes from segment L are highly expressed at 12 and 18 min. For phage phi6, RNA-dependent RNA polymerase within the phage particle transcribes S and M segments directly. However, the host protein YajQ is responsible for the activation of L transcription (Qiao et al., 2010b). Thus, we infer that segment S and M are immediately transcribed using RNA polymerase inside the phage particle and the initiation of L segment transcription might require a host protein(s), such as YajQ (PA4395), which results in delayed transcription of the L segment.

### Bacterial Transcriptomic Profile

**Figure 2:**
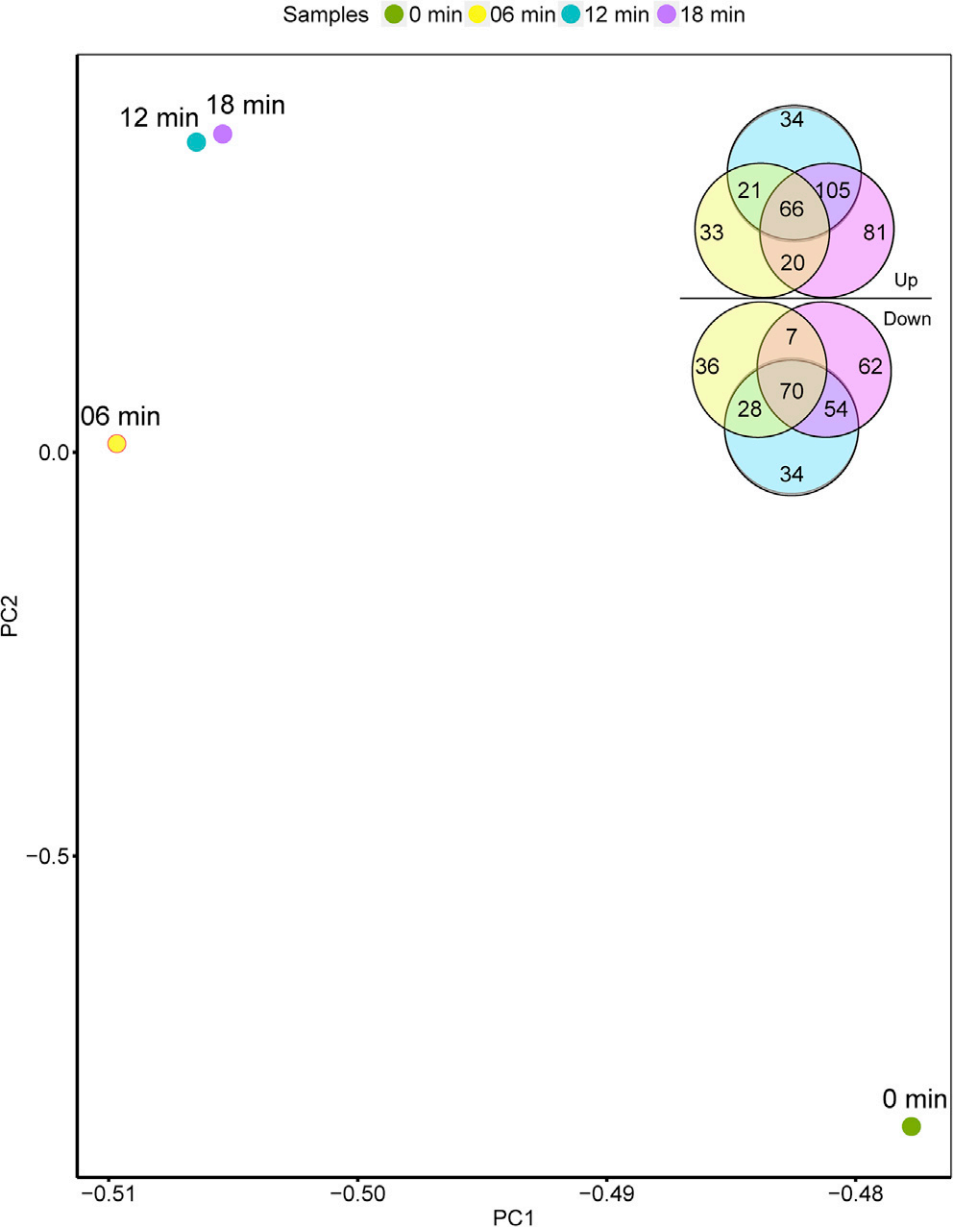
Bacterial DEGs Detected after Phage phiYY Infection at Three Time Points Totals of 5.37% (281/5,229), 7.88% (412/5,229), and 8.89% (465/5,229) DEGs were identified at 6, 12, and 18 min, respectively. The principal-component analysis was performed according to the expression abundance of PAO1r genes in phage-infected samples (6, 12, and 18 min) and phage-free samples (0 min). A greater distance between the four points indicates that the PAO1r gene expressions in the four samples are significantly different.

The bacterial gene expression level was measured by FPKM (Reads Per Kilobase Per Million Read), and genes with log2 fold change values of 1.5 and q values of <0.05 were defined as differentially expressed genes (DEGs) (Table S2). Totals of 5.37% (281/5,229), 7.88% (412/5,229), and 8.89% (465/5,229) DEGs were identified at 6, 12, and 18 min after phage infection, respectively (Figure 2). The majority of the host genes (87.55%, 4,578/5,229) are not significantly affected by phage, indicating a modest impact of dsRNA phage phiYY on host gene expression, compared with dsDNA phages (Yang et al., 2019).

RNA-seq data were validated by RT-qPCR. Fourteen DEGs were selected and the expression patterns were validated by RT-qPCR. The primers are listed in Table S3, and the RT-qPCR results are consistent with the RNA-seq results (Table 1).

**Table 1:** RT-qPCR Validation

Host Gene	Expression Fold Changes					
	RNA-Seq			RT-qPCR		
	6 min	12 min	18 min	6 min	12 min	18 min
*PA0499*	−7.235	−4.878	–	−3.153	−3.292	–
*PA1176*	−9.204	–	–	−5.897	–	–
*PA2849*	−2.968			−3.453	–	–
*PA3337*	−8.085	−11.150	−7.694	−3.026	−7.299	−8.960
*PA1562*	–	−3.315	−3.669		−3.225	−3.095
*PA1864*	−10.768	−11.763	−25.108	−3.205	−6.056	−11.834
*PA3879*	−5.178	−6.564	−7.127	−4.221	−6.887	−6.211
*PA4238*	–	–	3.010	–	–	2.322
*PA3609*	–	10.840	11.843	–	8.087	8.310
*PA5239*	4.160	3.332	–	2.745	2.303	–
*PA4745*	3.151			3.009	–	–
*PA2840*	6.4549	5.917	4.503	11.170	6.249	17.238
*PA0140*	–	55.407	22.816	–	16.986	8.389
*PA4613*	–	81.668	–	–	142.15	–

Seven downregulated genes and seven upregulated genes of *P. aeruginosa* were selected from the RNA-seq dataset and validated by RT-qPCR. The 16S rRNA gene was used as the reference gene for normalization. The gene expression fold change in RT-qPCR was determined by the delta-delta Ct method.

**Figure 3:**
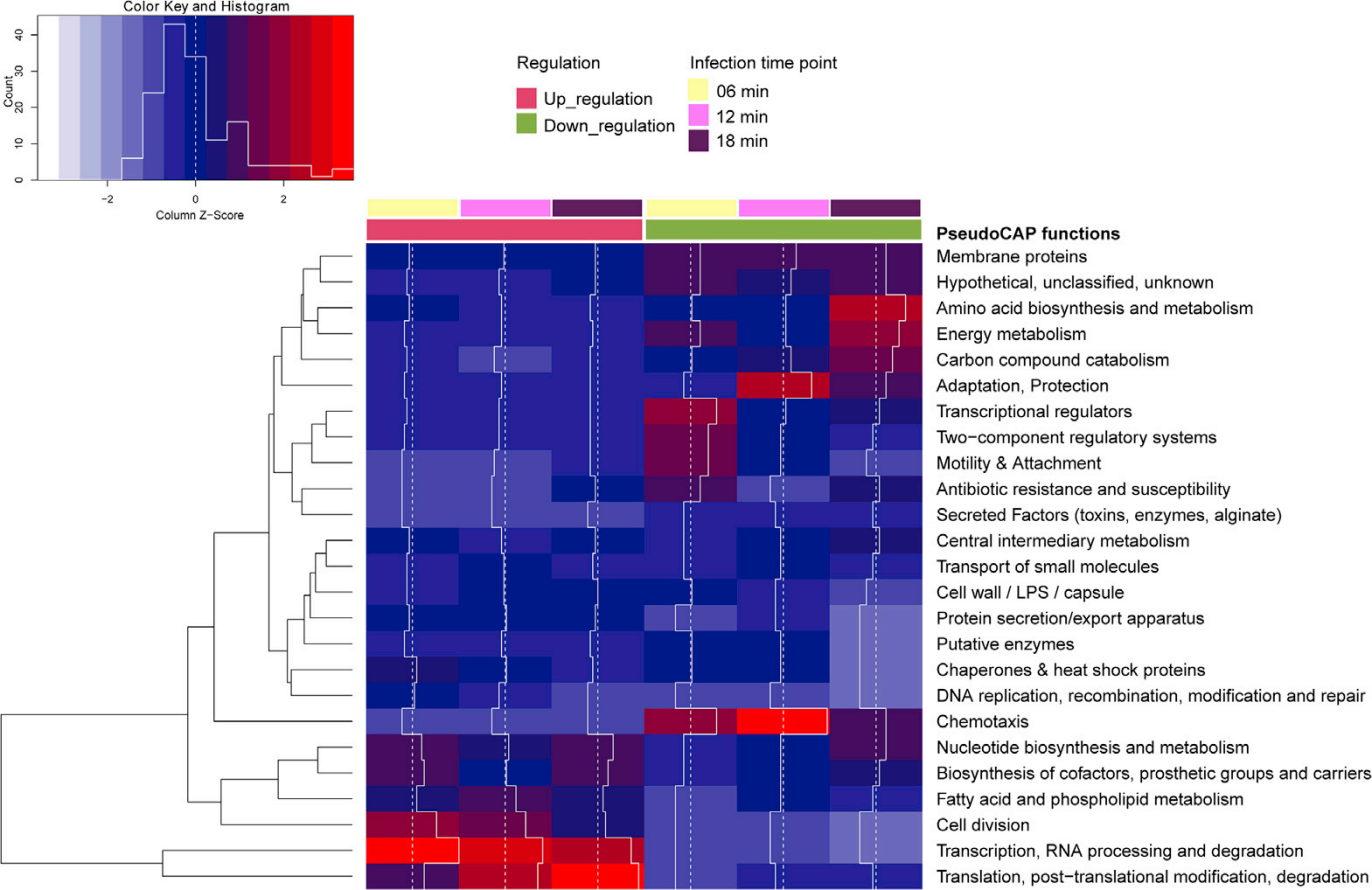
GO Analysis for the Biological Processes of DEGs During phiYY Infection The enriched upregulated and downregulated GO terms are shown. Most upregulated genes are involved in the pathways of transcription and translation. And the most downregulated genes are clustered as chemotaxis, transcriptional regulators, adaptation and protection, amino acid metabolism, and energy metabolism.

According to the gene ontology (GO) analysis, the host DEGs can be clustered into several groups (Figure 3). Most of the upregulated genes are involved in the pathways of transcription and translation, because these resources might be essential for dsRNA phage replication. Most downregulated genes are clustered as chemotaxis, transcriptional regulators, adaptation and protection, amino acid metabolism, energy metabolism, and carbon compound catabolism.

### Determine DEG Genes that Are Essential for dsRNA Phage Infection

We wanted to test whether the DEGs are expressed passively or are functional for either supporting or blocking phiYY life cycle. Thus, we tested the effects of the six most upregulated and six most downregulated DEGs on phiYY infection, by making single insertional deletions of these genes in PAO1r and testing the efficiency of plating (EOP) of phiYY on each mutant. Among the 12 mutants, *ΔPA4571, ΔPA3337* (*ΔrfaD*), and *ΔPA0848* (*ΔahpB*) are completely resistant to phiYY, and the deletion of *PA4613* (*katB*) decreased the EOP to 0.019 (Table 2).

**Table 2:** DEGs that Affect dsRNA Phage Infection Efficiency

Strain	Protein Function (Gene)	Expression Fold Change (log2)	EOP (p Value)
*ΔPA4571*	Cytochrome *c*	−3.09	No plaque
*ΔPA5170*	Arginine/ornithine antiporter (*arcD*)	−3.07	(1)
*ΔPA2754*	Hypothetical protein	−3.07	(1)
*ΔPA0545*	Hypothetical protein	−3.05	(1)
*ΔPA2247*	2-Oxoisovalerate dehydrogenase subunit alpha (*bkdA1*)	−3.02	(1)
*ΔPA3337*	ADP-L-glycero-D-mannoheptose-6-epimerase (*rfaD*)	−3.02	No plaque
*ΔPA0800*	Hypothetical protein	5.419	3.50 ± 1.07 (p < 0.05)
*ΔPA0140*	Alkyl hydroperoxide reductase (*ahpF*)	5.793	(1)
*ΔPA0849*	Thioredoxin reductase (*trxB2*)	6.031	(1)
*ΔPA4613*	Catalase(*katB*)	6.352	0.019 ± 0.03 (p < 0.05)
*ΔPA0848*	Alkyl hydroperoxide reductase (*ahpB*)	7.065	No plaque
*ΔPA3287*	Hypothetical protein	7.131	(1)

The six most downregulated and upregulated host genes affected by phiYY were selected. Each gene was knocked out in PAO1r by insertional deletion. EOP of phiYY on each knockout strain was measured by comparing with that of PAO1r. (1): no significant difference of the EOP.

Four of the six most upregulated genes are anti-oxidative stress genes, including alkyl hydroperoxide reductase (*PA0848* and *PA0140*), catalase (*PA4613*), and thioredoxin reductase (*PA0849*). And *ahpB* and *katB* are important for phiYY infection (Table 2), which indicates that the host-encoded, anti-oxidative stress pathway is essential for dsRNA phage replication. This is reasonable, because RNA molecules are more sensitive to oxidative damages than DNA molecules. For DNA phages, the deletion of anti-oxidative genes had a modest impact on phage infectivity. For example, *Campylobacter jejuni* phage NCTC 12673 has an EOP of 0.15 on a *katA* mutant compared with that of a wild-type bacterium (Sacher et al., 2018). Thus, we infer that host-encoded anti-oxidative stress machines are essential to protect phage RNA genomes from oxidative damage and are essential for dsRNA phage infection cycles.

Moreover, *PA0800* is highly expressed during phage infection and the deletion of *PA0800* increased the EOP to 3.5, indicating that this uncharacterized hypothetical protein might be involved in blocking phage infection. Although the function of PA0800 is unknown, its mechanism is a prospect for investigation in the near future.

For the most downregulated genes, four of six of the genes did not affect phage cycle, whereas *ΔPA4571* and *ΔPA3337* completely resisted phage infection. *PA3337* encodes an ADP-L-glycero-D-mannoheptose epimerase (*rfaD*), which is involved in the biosynthesis of the lipopolysaccharide precursor (Drazek et al., 1995). We performed adsorption assay to test the binding of phiYY to *ΔPA3337* and PAO1r. The adsorption rate to PAO1r was ~90%, whereas it decreased to ~10% for *ΔPA3337* (Figure S1). These data indicate that the deletion of *PA3337* blocks phage adsorption and results in a complete loss of plaques. But its downregulation during phage infection is intriguing, as it may be unable to deter phage infection, seeing as the phage has already entered the bacteria. One possible explanation is to prevent further phage infection, after the first phage entering, which is similar to superinfection exclusion (Abedon, 2015; Labrie et al., 2010). *PA4571* is annotated as a cytochrome *c* oxidase, and the mechanism of *PA4571* on phage infection is unknown.

These data indicate that DEGs are not expressed passively but are the results of complicated bacterial defense mechanisms and dsRNA phage-encoded hijacking mechanisms. Some DEGs might be bacterial defense approaches to constrain phiYY infection, such as *PA0800*. On the contrary, some DEGs might be controlled by phages to assist phage infection cycles, such as the highly expressed anti-oxidative stress genes.

Phages are viruses that are dependent on bacteria for replication. Thus, the host-encoded genes that are essential for dsRNA phage phi6, phi8, or phi2954 infection had been investigated previously. Mindich et al. have found that most of the cystoviridae family utilizes particular host proteins for the control of gene expression from the large genomic segment (Qiao et al., 2010b). Different dsRNA phages use YajQ, GrxC, GrxD, and Bcp for the induction of transcription of large L segments, which have guanine nucleotides missing at the first or second five prime positions relative to that found for segments S and M (Qiao et al., 2008, 2010a). Moreover, by screening the transposon mutant library, they found mutations affecting pilus formation or LPS composition to be of consequence for susceptibility to infection of different dsRNA phages, because pilus or LPS is the phage receptor (Qiao et al., 2010c).

However, in this study, we tested the effect of DEGs on phage infection cycle and found that some DEGs are essential for dsRNA phage infection, whereas PA0800 hinders phage infection. Since dsRNA phage phiYY only encodes 20 ORFs, it is reasonable to infer that dsRNA phage replication is massively dependent on host machinery.

### Bacteria-dsRNA Phage Interaction Network Analysis

**Figure 4:**
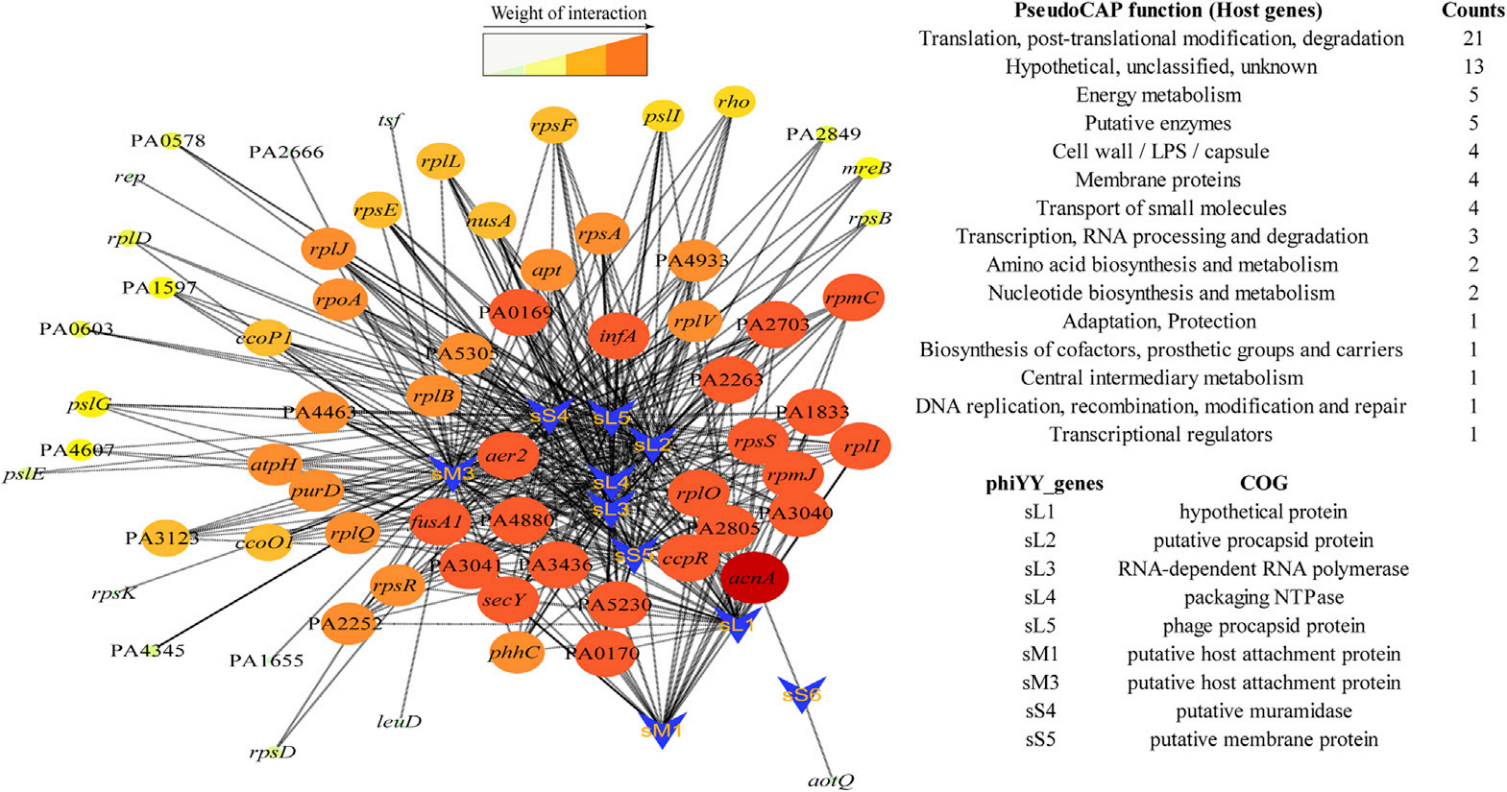
Gene Coexpression Network between PAO1r and phiYY Main networks of phage genes (blue arrow) and bacterial genes (oval) are shown and the size of the nodes indicates the interaction strength. Genes with more links are shown in bigger size, and the phage and host genes with high potential interactions are listed.

To predict the phage genes that might regulate host gene expression, the phage and bacterial gene coexpression analysis were conducted (Zhao et al., 2016), which identified nine phage genes that have expression pattern correlations with host genes (Figure 4). The results indicate that phage genes, such as putative procapsid protein, RNA-dependent RNA polymerase, and packing NTPase, may play a central role in interacting with host genes.

Many dsDNA phages encode proteins that could regulate the host gene expression (Roucourt and Lavigne, 2009). For example, T4 phage uses Alc protein to cut off host transcription (Kashlev et al., 1993). However, dsRNA phage phiYY has a limited genome with only 20 annotated genes, and most of these genes are involved in phage replication, packaging, attachment, and lysis (Yang et al., 2016), with eight genes that have yet to be characterized. To our knowledge, the impact of dsRNA phage-encoded proteins on host expression has yet to be studied. Thus, further studies of the phage genes might be interesting to test whether dsRNA phages could control and manipulate host gene expression and which phage protein(s) could upregulate the expression of anti-oxidative stress genes.

### Conclusion

This work represents the first RNA-seq analysis of the dsRNA phage infection processes, revealing dsRNA phage expression patterns and host responses. Furthermore, the phage-host interaction network analysis predicted some dsRNA phage-encoded proteins that may be involved in manipulating host gene expression. Finally, the host determinants of dsRNA phage susceptibility were investigated by studying the impact of significantly changed DEGs on phage infectivity, and the study reveals that phiYY is critically dependent on host machines to successfully replicate. These results raise new questions regarding host defense mechanisms against dsRNA phages and dsRNA phage-encoded hijacking mechanisms.

### Limitations of the Study

• The molecular mechanism of anti-oxidative genes on protecting phiYY infection has not been investigated yet.
• Further research is needed to elucidate whether dsRNA phage could control and manipulate host gene expression.

### Resource Availability

#### Lead Contact

Further information should be directed and will be fulfilled by the Lead Contact, Shuai Le (leshuai2004@tmmu.edu.cn).

#### Materials Availability

Available from the Lead Contact under the public/local regulation and Material Transfer Agreements (MTAs) for education and research purpose if they are used not for commercial purposes.

#### Data and Code Availability

The raw RNA-seq reads and analyzed files were deposited in the NCBI GEO: GSE128811.

## Methods

All methods can be found in the accompanying Transparent Methods supplemental file.

## Supplemental Information

Document S1. Transparent Methods and Figure S1

Table S1. Bacterial Strains and Phages Used in this Study, Related to Table 2

Table S2. DEGs Detected During phiYY Infection, Related to Figure 2

Table S3. Primers Used in this Study, Related to Tables 1 and 2

## References

[bib1] Abedon S.T. (2015). Bacteriophage secondary infection. Virol. Sin..

[bib2] Bao J., Wu N., Zeng Y., Chen L., Li L., Yang L., Zhang Y., Guo M., Li L., Li J. (2020). Non-active antibiotic and bacteriophage synergism to successfully treat recurrent urinary tract infection caused by extensively drug-resistant *Klebsiella pneumoniae*. Emerg. Microbes Infect..

[bib3] Ceyssens P.-J., Lavigne R. (2010). Bacteriophages of *Pseudomonas*. Future Microbiol..

[bib4] Ceyssens P.-J., Minakhin L., Van den Bossche A., Yakunina M., Klimuk E., Blasdel B., De Smet J., Noben J.-P., Blaesi U., Severinov K., Lavigne R. (2014). Development of giant bacteriophage phi KZ is independent of the host transcription apparatus. J. Virol..

[bib5] Chevallereau A., Blasdel B.G., De Smet J., Monot M., Zimmermann M., Kogadeeva M., Sauer U., Jorth P., Whiteley M., Debarbieux L., Lavigne R. (2016). Next-generation "-omics" approaches reveal a massive alteration of host RNA metabolism during bacteriophage infection of *Pseudomonas aeruginosa*. PLoS Genet..

[bib6] De Smet J., Zimmermann M., Kogadeeva M., Ceyssens P.-J., Vermaelen W., Blasdel B., Jang H.B., Sauer U., Lavigne R. (2016). High coverage metabolomics analysis reveals phage-specific alterations to *Pseudomonas aeruginosa* physiology during infection. ISME J..

[bib7] Drazek E.S., Stein D.C., Deal C.D. (1995). A mutation in the *Neisseria gonorrhoeae rfaD* homolog results in altered lipooligosaccharide expression. J. Bacteriol..

[bib8] Forti F., Roach D.R., Cafora M., Pasini M.E., Horner D.S., Fiscarelli E.V., Rossitto M., Cariani L., Briani F., Debarbieux L., Ghisotti D. (2018). Design of a broad-range bacteriophage cocktail that reduces *Pseudomonas aeruginosa* biofilms and treats acute infections in two animal models. Antimicrob. Agents Chemother..

[bib9] Hocquet D., Petitjean M., Rohmer L., Valot B., Kulasekara H.D., Bedel E., Bertrand X., Plesiat P., Kohler T., Pantel A. (2016). Pyomelanin-producing *Pseudomonas aeruginosa* selected during chronic infections have a large chromosomal deletion which confers resistance to pyocins. Environ. Microbiol..

[bib10] Jault P., Leclerc T., Jennes S., Pirnay J.P., Que Y.A., Resch G., Rousseau A.F., Ravat F., Carsin H., Le Floch R. (2018). Efficacy and tolerability of a cocktail of bacteriophages to treat burn wounds infected by *Pseudomonas aeruginosa* (PhagoBurn): a randomised, controlled, double-blind phase 1/2 trial. Lancet Infect. Dis..

[bib11] Kashlev M., Nudler E., Goldfarb A., White T., Kutter E. (1993). Bacteriophage T4 Alc protein: a transcription termination factor sensing local modification of DNA. Cell.

[bib12] Labrie S.J., Samson J.E., Moineau S. (2010). Bacteriophage resistance mechanisms. Nat. Rev. Microbiol..

[bib13] Lavigne R., Lecoutere E., Wagemans J., Cenens W., Aertsen A., Schoofs L., Landuyt B., Paeshuyse J., Scheer M., Schobert M., Ceyssens P.-J. (2013). A multifaceted study of *Pseudomonas aeruginosa* shutdown by virulent Podovirus LUZ19. mBio.

[bib14] Li T., Zhang Y., Dong K., Kuo C.J., Li C., Zhu Y.Q., Qin J., Li Q.T., Chang Y.F., Guo X., Zhu Y. (2020). Isolation and characterization of the novel phage JD032 and global transcriptomic response during JD032 infection of *Clostridioides difficile* ribotype 078. mSystems.

[bib15] Lood C., Danis-Wlodarczyk K., Blasdel B.G., Jang H.B., Vandenheuvel D., Briers Y., Noben J.P., van Noort V., Drulis-Kawa Z., Lavigne R. (2020). Integrative omics analysis of *Pseudomonas aeruginosa* virus PA5oct highlights the molecular complexity of jumbo phages. Environ. Microbiol..

[bib16] Mantynen S., Sundberg L.R., Oksanen H.M., Poranen M.M. (2019). Half a century of research on membrane-containing bacteriophages: bringing new concepts to modern virology. Viruses.

[bib17] Mantynen S., Sundberg L.R., Poranen M.M. (2018). Recognition of six additional cystoviruses: *Pseudomonas* virus phi6 is no longer the sole species of the family Cystoviridae. Arch. Virol..

[bib18] Nemecek D., Heymann J.B., Qiao J., Mindich L., Steven A.C. (2010). Cryo-electron tomography of bacteriophage phi6 procapsids shows random occupancy of the binding sites for RNA polymerase and packaging NTPase. J. Struct. Biol..

[bib19] Qiao X., Sun Y., Qiao J., Mindich L. (2008). The role of host protein YajQ in the temporal control of transcription in bacteriophage Phi6. Proc. Natl. Acad. Sci. U S A.

[bib20] Qiao J., Qiao X., Sun Y., Mindich L. (2010). Role of host protein glutaredoxin 3 in the control of transcription during bacteriophage Phi2954 infection. Proc. Natl. Acad. Sci. U S A.

[bib21] Qiao X., Sun Y., Qiao J., Mindich L. (2010). Interaction of a host protein with core complexes of bacteriophage phi6 to control transcription. J. Virol..

[bib22] Qiao X.Y., Sun Y., Qiao J., Di Sanzo F., Mindich L. (2010). Characterization of Phi 2954, a newly isolated bacteriophage containing three dsRNA genomic segments. BMC Microbiol..

[bib23] Rohde C., Resch G., Pirnay J.P., Blasdel B.G., Debarbieux L., Gelman D., Gorski A., Hazan R., Huys I., Kakabadze E. (2018). Expert opinion on three phage therapy related topics: bacterial phage resistance, phage training and prophages in bacterial production strains. Viruses.

[bib24] Roucourt B., Lavigne R. (2009). The role of interactions between phage and bacterial proteins within the infected cell: a diverse and puzzling interactome. Environ. Microbiol..

[bib25] Sacher J.C., Flint A., Butcher J., Blasdel B., Reynolds H.M., Lavigne R., Stintzi A., Szymanski C.M. (2018). Transcriptomic analysis of the *Campylobacter jejuni* response to T4-like phage NCTC 12673 infection. Viruses.

[bib26] Shen M., Zhang H., Shen W., Zou Z., Lu S., Li G., He X., Agnello M., Shi W., Hu F., Le S. (2018). *Pseudomonas aeruginosa* MutL promotes large chromosomal deletions through non-homologous end joining to prevent bacteriophage predation. Nucleic Acids Res..

[bib27] Shen M.Y., Le S., Jin X.L., Li G., Tan Y.L., Li M., Zhao X., Shen W., Yang Y.H., Wang J. (2016). Characterization and comparative genomic analyses of *Pseudomonas aeruginosa* phage PaoP5: new members assigned to PAK_P1-like viruses. Sci. Rep..

[bib28] Smith W.D., Bardin E., Cameron L., Edmondson C.L., Farrant K.V., Martin I., Murphy R.A., Soren O., Turnbull A.R., Wierre-Gore N. (2017). Current and future therapies for *Pseudomonas aeruginosa* infection in patients with cystic fibrosis. FEMS Microbiol. Lett..

[bib29] Vidaver A.K., Koski R.K., Van Etten J.L. (1973). Bacteriophage phi6: a lipid-containing virus of *Pseudomonas phaseolicola*. J. Virol..

[bib30] Waters E.M., Neill D.R., Kaman B., Sahota J.S., Clokie M.R.J., Winstanley C., Kadioglu A. (2017). Phage therapy is highly effective against chronic lung infections with *Pseudomonas aeruginosa*. Thorax.

[bib31] Waters V., Grimwood K. (2018). Defining chronic *Pseudomonas aeruginosa* infection in cystic fibrosis. J. Cyst. Fibros..

[bib32] Yang Y., Shen W., Zhong Q., Chen Q., He X., Baker J.L., Xiong K., Jin X., Wang J., Hu F., Le S. (2020). Development of a bacteriophage cocktail to constrain the emergence of phage-resistant *Pseudomonas aeruginosa*. Front. Microbiol..

[bib33] Yang Y.H., Lu S.G., Shen W., Zhao X., Shen M.Y., Tan Y.L., Li G., Li M., Wang J., Hu F.Q., Le S. (2016). Characterization of the first double-stranded RNA bacteriophage infecting *Pseudomonas aeruginosa*. Sci. Rep..

[bib34] Yang Z., Yin S., Li G., Wang J., Huang G., Jiang B., You B., Gong Y., Zhang C., Luo X. (2019). Global transcriptomic analysis of the interactions between phage phiAbp1 and extensively drug-resistant *Acinetobacter baumannii*. mSystems.

[bib35] You L., Shi J., Shen L., Li L., Fang C., Yu C., Cheng W., Feng Y., Zhang Y. (2019). Structural basis for transcription antitermination at bacterial intrinsic terminator. Nat. Commun..

[bib36] Zhao X., Chen C., Jiang X., Shen W., Huang G., Le S., Lu S., Zou L., Ni Q., Li M. (2016). Transcriptomic and metabolomic analysis revealed multifaceted effects of phage protein Gp70.1 on *Pseudomonas aeruginosa*. Front. Microbiol..

[bib37] Zhao X., Shen M., Jiang X., Shen W., Zhong Q., Yang Y., Tan Y., Agnello M., He X., Hu F., Le S. (2017). Transcriptomic and metabolomics profiling of phage-host interactions between phage PaP1 and *Pseudomonas aeruginosa*. Front. Microbiol..

