# Supplementary material for: Transcriptomic Analysis Reveals the Dependency of Pseudomonas aeruginosa Genes for Double-Stranded RNA Bacteriophage phiYY Infection Cycle
Source: iScience. 2020 Aug 6;23(9):101437. doi: 10.1016/j.isci.2020.101437 (PMC7452160; doi:10.1016/j.isci.2020.101437)
Supplement: Document S1. Transparent Methods and Figure S1 [file mmc1.pdf]

iScience, Volume *23*

Supplemental Information

Transcriptomic Analysis Reveals the Dependency of *Pseudomonas aeruginosa* Genes for Double-Stranded RNA Bacteriophage phiYY Infection Cycle

Qiu Zhong, Lan Yang, Linlin Li, Wei Shen, Yang Li, Huan Xu, Zhuojun Zhong, Ming Chen, and Shuai Le

Supplemental Information

Transcriptomic analysis reveals the dependency of *Pseudomonas aeruginosa* genes for double-stranded RNA bacteriophage phiYY infection cycle

Qiu Zhong[1,2], Lan Yang[3], linlin Li[3], Wei Shen[4], Yang Li[5,6], Huan Xu[2], Zhuojun Zhong[4], Ming Chen[2,6*], Shuai Le[4,7*]

[1] Department of Clinical Laboratory Medicine, Daping Hospital, Army Medical University, Chongqing, 400038, China

[2] Department of Clinical Laboratory Medicine, Southwest Hospital, Army Medical University, Chongqing , 400038,China

[3] Shanghai Institute of Phage, Shanghai Public Health Clinical Center, Fudan University, Shanghai, 201508, China

[4] Department of Microbiology, College of Basic Medical Sciences, Army Medical University, Chongqing, 400038,China

[5] Medical Center of Trauma and War injury, Daping Hospital, Army Medical University, Chongqing, 400038,China

[6] State Key Laboratory of Trauma, Burns and Combined Injuries, Army Medical University, Chongqing, 400038, China

[7] Lead Contact

* To whom correspondence should be addressed:
Shuai Le, leshuai2004@tmmu.edu.cn;
Ming Chen, chming1971@126.com;

Transparent Methods

1. Bacterial strains, phages and culture conditions.

The bacterial strains and phages in this work are listed in Table S1. Phage phiYY was previously isolated from the sewage of Southwest Hospital in Chongqing, China. The accession numbers of phiYY genome segments were deposited in GenBank (Yang *et al.*, 2016). (Segments L, M, and S are KX074201, KX074202, and KX074203, respectively). The previously sequenced PAO1r_8 was referred in short as PAO1r in this study (Shen *et al.*, 2018). PAO1r lost 341 genes (*PA1880-PA2220*), including *galU* and therefore lost O-antigen. *P. aeruginosa* strains were grown in Luria-Bertani (LB) broth at 37°C. When required, gentamycin (25μg/ml) were used in LB broth.

2. One-step growth curve of phiYY on PAO1r

One-step growth curve of phiYY was performed as previously described (Yang *et al.*, 2019). PAO1r was infected with phiYY with an MOI (multiplicity of infection) of 0.1. After a 5 min adsorption, the mixed culture was centrifuged for 1 min at 12,000×g. It was then washed twice with LB medium to remove the unadsorbed phages. The pellets were then resuspended in 5ml of LB medium. After which the culture was diluted 10,000 fold to limit post-adsorption of uninfected bacteria. Then, the cultures were grown at 37°C with shaking. Samples were taken every 10 min until 80 min after phage infection. For each time point, the samples were pelleted immediately for 30 s at 12,000 ×g, and the phage titer in the supernatant was determined by double-layer agar plaque method. Three biological repeats were performed.

3. Preparing samples for RNA-seq and RT-qPCR

10 ml of bacterial PAO1r culture (OD600=0.5) was infected with phage phiYY at an MOI of 10, and 1 ml of uninfected sample was taken as the negative control (0 min sample). The infected culture was grown at 37°C with shaking. 1 ml culture was taken from the infected culture at three time points (6, 12 and 18min) for RNA extraction. Three biological repeats were performed.

4. RNA extraction and RNA-seq analysis.

RNA extraction and RNA-seq analysis were performed as previously described (Li *et al.*, 2020). Briefly, total RNA was extracted from each sample (0, 6, 12, and 18 min after phage infection) by SV Total RNA Isolation System (Promega, USA). RNA quality and quantity were checked using Bioanalyzer (Anilent, USA) and RNA 6000 Nano kit (Anilent, USA) .For RNA-seq experiments, rRNA was removed using the Ribo-Zero rRNA removal kit. The cDNA libraries were constructed and sequenced on an Illumina HiSeq 2500 sequencer (Illumina, USA), using paired-end 2- by 150-bp reads.

Bowtie 2 was used to map the reads to PAO1r and phiYY genome, respectively (Langmead & Salzberg, 2012). Tophat2 and Cufflinks 2.2.1 were used to analyze the RNA-seq data and identify differential genes (Trapnell *et al.*, 2012, Kim *et al.*, 2013). The Reads Per Kilobase Per Million Read (FPKM) and the false discovery rate (FDR) (q value) were used to determined gene expression changes. Differentially expressed genes (DEGs) between the two groups were calculated by DESeq, and DEGs were determined with a log2 fold change value of 1.5 and a q value of 0.05.

GO term enrichment of DEGs was analyzed by Blast2GO software (BioBam). And KEGG pathway was clustered by KOBAS (Xie *et al.*, 2011) (http://kobas.cbi.pku.edu.cn/).The gene coexpression networks were analyzed and visualized using Cytoscape3.4.0 (https://cytoscape.org).

5. RT-qPCR validation.

13 DEGs were selected for RT-qPCR validation. RT-qPCR was performed using SYBR Premix Ex Taq II (TaKaRa Bio, China). The primers used in this study are listed in Table S3. The 16S rRNA gene was used as the reference gene for normalization, and the expression of each gene was compared with that of the uninfected host using the delta-delta Ct method.

6. Insertion deletion of the 12 DEGs in PAO1r

12 insertional mutants were constructed by a previously described procedure (Le *et al.*, 2014). Briefly, to knock out *PA5471* in PAO1r, a small fragment within *PA5471* was amplified by primers 5471-KO-F and 5471-KO-R (Table S3), using PAO1r genome as template. The PCR fragment was digested with EcoRI/BamHI, and ligated into the EcoRI/BamHI digested plasmid pEX18Gm, resulting in pEX-5471. Then, pEX-5471 was electroporated into PAO1r to generate an insertional mutant via a single crossover. The other 11 knock out mutants were generated with the primers listed in Table S3.

7. EOP experiment and statistical analysis

The EOP experiment of phiYY on 12 insertional deletion mutants was determined by double-layer agar plate assay. Briefly, 10 μl of serial 10-fold dilutions of phiYY was mixed with 200 μl host, and mixed with 5 ml of 0.7% LB agar, and poured on LB agar plate. The phage titer on each strain was calculated after observation of plaques after overnight incubation, and three biological repeats were performed. EOP was calculated as phage titer on host strain divided by phage titer on PAO1r. The statistical analysis was performed using student's t test, and a P value < 0.05 was considered as statistically significant.

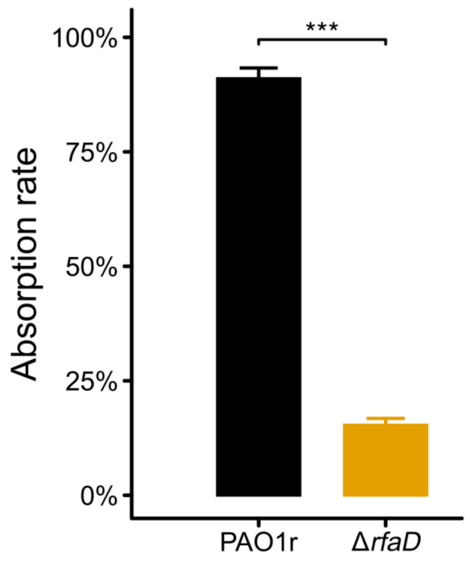

Figure S1 Adsorption assay of bacteriophages phiYY to PAO1r and Δ *rfaD*. Related to Table2. Percent adsorption of the phage was calculated as [(initial titer − residual titer) / initial titer] × 100%. There biological repeats were performed. The asterisks mark *P*-value of < 0.05 as calculated by Student's t-test.

References

Kim, D., G. Pertea, C. Trapnell, H. Pimentel, R. Kelley & S.L. Salzberg, (2013) TopHat2: accurate alignment of transcriptomes in the presence of insertions, deletions and gene fusions. *Genome Biol* **14**: R36.

Langmead, B. & S.L. Salzberg, (2012) Fast gapped-read alignment with Bowtie 2. *Nature methods* **9**: 357-359.

Le, S., X. Yao, S. Lu, Y. Tan, X. Rao, M. Li, X. Jin, J. Wang, Y. Zhao, N.C. Wu, R. Lux, X. He, W. Shi & F. Hu, (2014) Chromosomal DNA deletion confers phage resistance to *Pseudomonas aeruginosa*. *Scientific reports* **4**: 4738.

Li, T., Y. Zhang, K. Dong, C.J. Kuo, C. Li, Y.Q. Zhu, J. Qin, Q.T. Li, Y.F. Chang, X. Guo & Y. Zhu, (2020) Isolation and Characterization of the Novel Phage JD032 and Global Transcriptomic Response during JD032 Infection of *Clostridioides difficile* Ribotype 078. *mSystems* .5(3):e00017-20.

Shen, M., H. Zhang, W. Shen, Z. Zou, S. Lu, G. Li, X. He, M. Agnello, W. Shi, F. Hu & S. Le, (2018) *Pseudomonas aeruginosa* MutL promotes large chromosomal deletions through non-homologous end joining to prevent bacteriophage predation. *Nucleic Acids Res* **46**: 4505-4514.

Trapnell, C., A. Roberts, L. Goff, G. Pertea, D. Kim, D.R. Kelley, H. Pimentel, S.L. Salzberg, J.L. Rinn & L. Pachter, (2012) Differential gene and transcript expression analysis of RNA-seq experiments with TopHat and Cufflinks. *Nature protocols* **7**: 562-578.

Xie, C., X. Mao, J. Huang, Y. Ding, J. Wu, S. Dong, L. Kong, G. Gao, C.Y. Li & L. Wei, (2011) KOBAS 2.0: a web server for annotation and identification of enriched pathways and diseases. *Nucleic Acids Res* **39**: W316-322.

Yang, Y.H., S.G. Lu, W. Shen, X. Zhao, M.Y. Shen, Y.L. Tan, G. Li, M. Li, J. Wang, F.Q. Hu & S. Le, (2016) Characterization of the first double-stranded RNA bacteriophage infecting *Pseudomonas aeruginosa. Scientific reports* 9;6:38795.

Yang, Z., S. Yin, G. Li, J. Wang, G. Huang, B. Jiang, B. You, Y. Gong, C. Zhang, X. Luo, Y. Peng & X. Zhao, (2019) Global Transcriptomic Analysis of the Interactions between Phage phiAbp1 and Extensively Drug-Resistant Acinetobacter baumannii. *mSystems* 16;4(2):e00068-19.
