# Supplementary material for: Transcriptomic Analysis Reveals the Dependency of Pseudomonas aeruginosa Genes for Double-Stranded RNA Bacteriophage phiYY Infection Cycle
Source: iScience. 2020 Aug 6;23(9):101437. doi: 10.1016/j.isci.2020.101437 (PMC7452160; doi:10.1016/j.isci.2020.101437)
Supplement: Table S1. Bacterial Strains and Phages Used in this Study, Related to Table 2 [file mmc2.docx]

**TABLE S1 Bacterial strains and phages used in this study. Related to table2**

| Strain or phage | Description | Source |
| --- | --- | --- |
| PAO1r | PAO1*ΔPA1880-PA2220* | (Shen *et al*., 2018) |
| phiYY | dsRNA bacteriophage | (Yang *et al*., 2016) |
| *ΔPA4571* | PAO1r*ΔPA4571* | This study |
| *ΔPA5170* | PAO1r*ΔPA5170* | This study |
| *ΔPA2754* | PAO1r*ΔPA2754* | This study |
| *ΔPA0545* | PAO1r*ΔPA0545* | This study |
| *ΔPA2247* | PAO1r*ΔPA2247* | This study |
| *ΔPA3337* | PAO1r*ΔPA3337* | This study |
| *ΔPA0800* | PAO1r*ΔPA0800* | This study |
| *ΔPA0140* | PAO1r*ΔPA0140* | This study |
| *ΔPA0849* | PAO1r*ΔPA0849* | This study |
| *ΔPA4613* | PAO1r*ΔPA4613* | This study |
| *ΔPA0848* | PAO1r*ΔPA0848* | This study |
| *ΔPA3287* | PAO1r*ΔPA3287* | This study |
