# Supplementary material for: Transcriptomic Analysis Reveals the Dependency of Pseudomonas aeruginosa Genes for Double-Stranded RNA Bacteriophage phiYY Infection Cycle
Source: iScience. 2020 Aug 6;23(9):101437. doi: 10.1016/j.isci.2020.101437 (PMC7452160; doi:10.1016/j.isci.2020.101437)
Supplement: Table S3. Primers Used in this Study, Related to Tables 1 and 2 [file mmc4.docx]

**Table S3 Primers used in this study. Related to Table1 and Table2.**

| **Primer** | **Sequence (5’ to 3’)** | **Description** |
| --- | --- | --- |
| PA0499-F | GAAGCCATCGAACACCTGAT | RT-qPCR |
| PA0499-R | CGTTGGTCTCTACGCCTGAT |  |
| PA1176-F | ATGAGCAGTCGCCGAGAG |  |
| PA1176-R | GAGAGGTCGAGTTCGACGAG |  |
| PA2849-F | AGGGCGAAGGTCTGATTACC |  |
| PA2849-R | CAGCAGCTCATCCTTTAGGG |  |
| PA3337-F | CCGCTACATGATGGAGAACA |  |
| PA3337-R | GCTGGTCGAAGAGGAACTTG |  |
| PA1562-F | TGGACAGCCTGAAAACCCTT |  |
| PA1562-R | AGCAGGACCTTCAGCGACAT |  |
| PA1864-F | GAAGGCCAACGTGCTCTATT |  |
| PA1864-R | TGATCTCGCAGGAGAAGACC |  |
| PA3879-F | CTGAACATGAAGGGCATGAA |  |
| *PA3879-R* | CTCCATGTCCTTGAGCAGGT |  |
| *PA4238-F* | AGATCGACGGCGTACTCCAC |  |
| *PA4238-R* | TATCGGCAGCAGTCACAACAC |  |
| *PA3609-F* | GACTGGTACCGCAAGCTGTT |  |
| *PA3609-R* | TTCAGGCCGATGAAGAAGAT |  |
| *PA5239-F* | ACCGAACTCAAGCAAAAGCC |  |
| *PA5239-R* | AGAATCTCCAGCACGCCATC |  |
| *PA4745-F* | GCGAGGTCATCGAAGAAAAG |  |
| *PA4745-R* | GGGTGACCTTCTTCACGGTA |  |
| *PA2840-F* | ACCGCCTGCGAAACCTATT |  |
| *PA2840-R* | GAAGCGCCTCGAAGATCACT |  |
| *PA0140-F* | GGTGTACAAGGACCGCAACT |  |
| *PA0140-R* | GCGATCACGATCTGCTTGTA |  |
| *PA4613-F* | GACCCTGCTGTATTCCAACGA |  |
| *PA4613-R* | TGGACTTCAGCCACCTGTTTC |  |
| *16s-F* | CAAAAGCTACTGAGCTAGAGTACG |  |
| *16s-R* | TAAGATCTCAAGGATCCCAACGGCT |  |
| *PA2754-KO-F* | AAGAATTCTGAACAGATCAATTCCGTGCTGC | Insertion deletion |
| *PA2754-KO-R* | AAGGATCCACGTATTCCTCGGTCGCTTCG |  |
| *PA2247-KO-F* | AAGAATTCGGAGGCAGGTTTCTTCTCCATCTC |  |
| *PA2247-KO-R* | AAGGATCCGCGGTACTTGGACGGGTCGT |  |
| *PA3287-KO-F* | AAGAATTCTACGCCTGCTGCTGGCCTACAA |  |
| *PA3287-KO-R* | AAGGATCCCCGCCATCATCAGTGCGGTTTT |  |
| *PA0849-KO-F* | AAGGATCCGACGCACTGATCGTCGC |  |
| *PA0849-KO-R* | AAGAATTCCGGCGTTGAGCTTGAG |  |
| *PA4571-KO-F* | AAGGATCCCTGAAGTCCCTGCCGG |  |
| *PA4571-KO-R* | AAGAATTCCTTCCAGCTGGGCGA |  |
| *PA5170-KO-F* | AAGGATCCATCATGTTCGCCGCC |  |
| *PA5170-KO-R* | AAGAATTCCGATGACCAGGTCCTTGC |  |
| *PA0545-KO-F* | AAGGATCCTCGCCAGCGCCGACC |  |
| *PA0545-KO-R* | AAGAATTCAAGCGCCCGGCGAAC |  |
| *PA3337-KO-F* | AAGGATCCCCCGCGCCACGTCCT |  |
| *PA3337-KO-R* | AAGAATTCCGGACCTTCCGCGAG |  |
| *PA0800-KO-F* | AAGGATCCTAATTGAGATACAGTCGCAAACCT |  |
| *PA0800-KO-R* | AAGAATTCACCCACACGGCGAAG |  |
| *PA0140-KO-F* | AAGGATCCGGGCATTCGCACCGG |  |
| *PA0140-KO-R* | AAGAATTCCTCGCCGGGCACGCC |  |
| *PA4613-KO-F* | AAGGATCCTGCTCGCGCTGGATC |  |
| *PA4613-KO-R* | AAGAATTCCGTCGAACCTGGTGC |  |
| *PA0848-KO-F* | AAGGATCCTTTCGTCTGCCCCTCGG |  |
| *PA0848-KO-R* | AAGAATTCCCCCAGCGGCAGGTT |  |
